# Stanniocalcin Protein Expression in Female Reproductive Organs: Literature Review and Public Cancer Database Analysis

**DOI:** 10.1210/endocr/bqae110

**Published:** 2024-08-26

**Authors:** Masuma Khatun, Vijayachitra Modhukur, Terhi T Piltonen, Juha S Tapanainen, Andres Salumets

**Affiliations:** Department of Obstetrics and Gynecology, University of Helsinki and Helsinki University Hospital, Haartmaninkatu 8, 00290 Helsinki, Finland; Department of Obstetrics and Gynecology, Institute of Clinical Medicine, University of Tartu, 50406 Tartu, Estonia; Competence Centre on Health Technologies, 50411 Tartu, Estonia; Department of Obstetrics and Gynecology, Research Unit of Clinical Medicine, Medical Research Center, Oulu University Hospital, University of Oulu, 90220 Oulu, Finland; Department of Obstetrics and Gynecology, University of Helsinki and Helsinki University Hospital, Haartmaninkatu 8, 00290 Helsinki, Finland; Department of Obstetrics and Gynaecology, HFR—Cantonal Hospital of Fribourg and University of Fribourg, 79085 Fribourg, Switzerland; Department of Obstetrics and Gynecology, Institute of Clinical Medicine, University of Tartu, 50406 Tartu, Estonia; Competence Centre on Health Technologies, 50411 Tartu, Estonia; Division of Obstetrics and Gynecology, Department of Clinical Science, Intervention and Technology, Karolinska Institutet and Karolinska University Hospital, 14152 Huddinge, Stockholm, Sweden

**Keywords:** stanniocalcin, endometriosis, polycystic ovary syndrome, endometrial cancer, ovarian cancer, cervical cancer

## Abstract

Stanniocalcin (STC) 1 and 2 serve as antihyperglycemic polypeptide hormones with critical roles in regulating calcium and phosphate homeostasis. They additionally function as paracrine and/or autocrine factors involved in numerous physiological processes, including female reproduction. STC1 and STC2 contribute to the pathophysiology of several diseases, including female infertility- and pregnancy-associated conditions, and even tumorigenesis of reproductive organs. This comprehensive review highlights the dynamic expression patterns and potential dysregulation of STC1 and STC2, restricted to female fertility, and infertility- and pregnancy-associated diseases and conditions, such as endometriosis, polycystic ovary syndrome (PCOS), abnormal uterine bleeding, uterine polyps, and pregnancy complications, like impaired decidualization, preeclampsia, and preterm labor. Furthermore, the review elucidates the role of dysregulated STC in the progression of cancers of the reproductive system, including endometrial, cervical, and ovarian cancers. Additionally, the review evaluates the expression patterns and prognostic significance of STC in gynecological cancers by utilizing existing public datasets from The Cancer Genome Atlas to help decipher the multifaceted roles of these pleiotropic hormones in disease progression. Understanding the intricate mechanisms by which STC proteins influence all these reviewed conditions could lead to the development of targeted diagnostic and therapeutic strategies in the context of female reproductive health and oncology.

Human stanniocalcin-1 (STC1) is a homodimeric glycoprotein consisting of 247 amino acids, located on chromosome 8p21.2, that is 92% similar to the originally identified fish STC (1, 2). Conversely, human STC2 is a larger protein comprising 302 amino acids, located on chromosome 5q35.1, that is only 34% similar to human and fish STC1 (3, 4). Both STC1 and STC2 play diverse, pleiotropic roles in numerous physiological processes, such as calcium phosphate homeostasis (5, 6), bone remodeling (7, 8), organogenesis (9), angiogenesis (10, 11), retinal degeneration (12, 13), cellular proliferation (14, 15), apoptosis (16, 17), cerebral ischemia (18, 19), tumorigenesis (20, 21), inflammation (22, 23), antioxidative function (19, 24), and cellular metabolism (25, 26). Notably, mammalian STC controls somatic growth by locally blocking insulin-like growth factor (IGF) signaling (27, 28). Consequently, IGF binds to the IGF1 receptor, initiating downstream signaling cascades, such as PI3K–AKT–mTOR and RAS/RAF/MAPK pathways (29, 30). However, STC1 is barely detectable in serum, indicating its function as a paracrine/autocrine signaling molecule rather than a classic endocrine hormone in mammals (31, 32).

Mammalian STC1 exhibits a broad expression pattern, including in the endocrine glands and hormone-responsive organs, with peak levels found in the human ovaries, kidneys, prostate, and thyroid tissue (33, 34). Additionally, numerous studies have elucidated the significant role of STC, particularly STC1, in regulating both normal physiological processes and pathophysiological conditions within female reproductive tissue, such as the ovaries, uterus, and placenta (34). These studies, which were predominantly conducted on animal models, have shed light on critical aspects of female reproductive biology, including ovarian follicle maturation (35, 36), blastocyst implantation (37, 38), placental maturation (39, 40), vascularization during early pregnancy (41, 42), and lactation (43, 44). Conversely, STC expression is nearly undetectable in the testis (45), emphasizing its specific involvement in female reproduction (34). Moreover, the localization and distribution of STC1 receptors within ovarian tissues, primarily identified in animal models, particularly in luteal cells, underscore the potential role of STC1 in female reproductive physiology (45, 46).

Numerous studies have also explored the intriguingly complex roles of both STC1 and STC2, identifying them as potential universal tumor biomarkers and promising therapeutic targets across various cancers, including those affecting female reproductive tissues (47, 48). The expression pattern of STC is often characterized by overexpression in numerous cancers, which correlates with advanced disease stages and poor survival rates (49, 50). However, some studies have indicated a tumor-suppressive role for STC1 since the loss of function of this protein disrupts normal physiological activity, particularly in gynecological malignancies (51, 52). Regulation of *STC1* expression involves a complex network of transcription factors and signal transduction pathways, implying its role as an intermediate modulator rather than a precise cancer determinant factor (20). Hypoxia-inducible factor-1 (HIF-1) expression may modulate *STC1* under hypoxic conditions, initiating the expression of several downstream genes associated with metabolic reprogramming by activating the Warburg effect (53, 54). STC1 has also been implicated in various cancer-related signaling pathways, including NF-kB, ERK1/2, JNK, and epithelial–mesenchymal transition (EMT), contributing to tumor initiation, invasion, metastasis, inflammation, and therapeutic resistance (55-57). Besides, clinical data have shown a negative correlation between STC1 expression levels and prognosis, with higher levels associated with shorter disease-free and overall survival (OS). This underscores the potential of STC1 as a diagnostic, prognostic, and therapeutic target in cancer (20, 58, 59). In contrast, human STC2 is also implicated in tumor progression and metastasis, influenced significantly by hypoxia-induced endoplasmic reticulum stress in the tumor microenvironment (60-62). Moreover, elevated STC2 is associated with poor outcomes in pancreatic, nasopharyngeal, ovarian, colon, and lung cancers, suggesting its role as a prognostic marker although, its role in breast cancer is controversial (63-65). An overview of the existing literature on the expression pattern and postulated role of STC within female reproductive pathologies, including human cancers, is outlined in Table 1. The plausible regulatory network and impact of STC dysfunction on various gynecological pathologies are depicted in Fig. 1.

**Figure 1.**
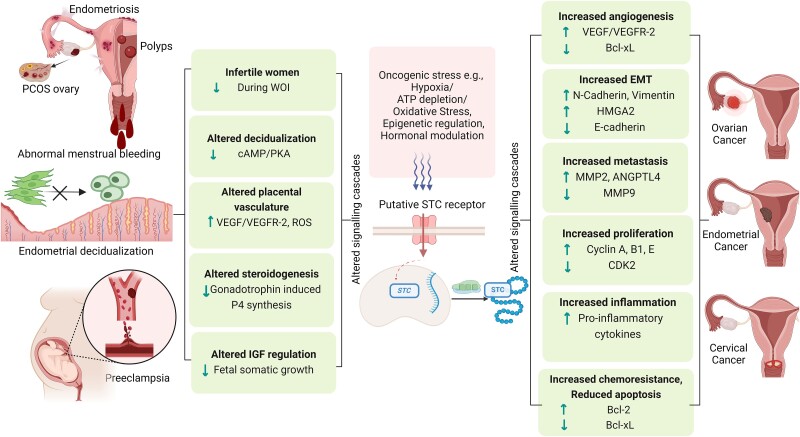
Regulation networks of stanniocalcin (STC) in gynecological disorders and tumor progression. The regulation networks governing STC involve a complex interplay of numerous proteins affecting both STC1 and STC2. These proteins intricately modulate various signaling pathways, exerting significant influence over gynecological disorders and tumor progression. Abbreviations: PCOS, polycystic ovarian syndrome; WOI, window of implantation; cAMP, cyclic adenosine monophosphate; PKA, protein kinase A; VEGF, vascular endothelial growth factor; VEGFR-2, vascular endothelial growth factor receptor 2; ROS, reactive oxygen species; P4, progesterone, IGF, insulin-like growth factor; Bcl, B-cell lymphoma; EMT, epithelial–mesenchymal transition; HMGA2, high mobility group AT-hook 2; MMP, matrix metalloproteinase; ANGPTL4, angiopoietin-like 4; CDK2, cyclin-dependent kinase 2. Figure created with BioRender.

**Table 1.** A summary of the expression pattern and localization of stanniocalcin (STC) genes and proteins in gynecological pathologies and cancer

Type	Pathology	Material/localization (gene, protein)	Regulation	Signaling pathways	Possible effect on condition/tumor	References
STC1	Idiopathic infertility	Endometrial biopsies from infertile women who conceived after ICSI Blood and eSCs from infertile women undergoing IVF with intrauterine PRP infusion	Up	Not reported	Improved decidualization Reduced inflammation	(66, 67)
STC1	Endometriosis	Endometrial biopsy tissue, eSCs, uterine fluid	Down	cAMP/PKA	Defective decidualization	(68)
STC1	PCOS	Endometrial biopsy tissue, eSCs	Down	cAMP/PKA	Compromised stress tolerance Defective decidualization	(69)
STC1	Abnormal uterine bleeding	Endometrial biopsy tissue, eSCs	Up	Angiogenesis/Extracellular matrix-linked	Modulate angiogenesis or vessel integrity	(70)
STC1 and 2	Uterine polyps	Endometrial biopsy tissue	Up	Not reported	Polyp formation in infertile women	(71)
STC1	Preeclampsia-linked, pregnancy complications	Plasma from preeclamptic pregnant women Placental syncytiotrophoblast, cytotrophoblast, endothelial, stromal cells, blood BeWo cell line	Up	cAMP/PKA/PI3 K/AKT/SGK-1	Placental development linked to hypoxia	(39, 40, 72)
STC1	Placental vasculature	Myometrial biopsies from term pregnant women Human vascular smooth muscle cell line, endothelial cell line, extravillous trophoblast cell line	Up	Not reported	Spiral artery remodeling	(41)
STC1 and 2	Preterm labor	Placental miRNA target gene	Up	Not reported	Adverse placental development	(73)
STC1	Ovarian cancer	Clinical, Serum, TMA OvCa tissue Transgenic mice, human omentum with OvCa CaOV3, OVCAR3, OVCA420, OVCA432, OVCA433, SKOV3, HEY, SKOV3-ip1, KURAMOCHI, OVCAR5, TYK-nu cell lines	Up	ITGβ6/PI3 K/FOXC2, Hypoxia linked	Aggressive OvCa progression Inhibit apoptosis Poor OS survival	(53, 55, 74-78)
STC2	Ovarian cancer	Clinical OvCa tissue SKOV3, T29, Caov-3 cell line	Up	EMT, ROS, ERK1/2	Aggressive OvCa progression Poor OS survival	(79, 80)
STC1	Endometrial cancer	Clinical TMA EnCa tissue	Down	Not reported	Aggressive EnCa progression No association with DFS	(81, 82)
STC2	Endometrial cancer	Clinical EnCa tissue Ishikawa and RL95-2 cell lines	Up	E2-dependent AMPK	Linked to grade 2-3 tumors Promote cellular proliferation and inhibiting apoptosis (type I EnCa) Poor RFS	(83, 84)
STC1	Cervical cancer	Clinical CeCa tissue, TMA CeCa tissue CaSki, HeLa cell line	Down	NF-κB phosphor-P65 (Ser536) regulated by PI3 K/AKT, IκBα, and IKK	Aggressive CeCa progression Linked to advanced-stage metastasis Inhibit apoptosis	(15, 85, 86)
STC2	Cervical cancer	Clinical CeCa tissue HT-3, HeLa cell line	Up	MAPK	Aggressive CeCa progression Detected in cisplatin-resistant cases Inhibit apoptosis Poor OS	(87, 88)

Abbreviations: AMPK, AMP-activated protein kinase; cAMP, cyclic adenosine monophosphate; CeCa, cervical cancer; DFS, disease-free survival; E2, estradiol; EMT, epithelial–mesenchymal transition; EnCa, endometrial cancer; ERK1/2, extracellular signal-regulated kinase 1/2; eSCs, endometrial stromal cells; FOXC2, Forkhead box protein C2; ICSI, intracytoplasmic sperm injection; IKK, nuclear factor-κB (IκB) kinase; ITGβ6, β6 integrin; IVF, in vitro fertilization; MAPK, mitogen-activated protein kinase; OS, overall survival; OvCa, ovarian cancer; PI3K, phosphoinositide 3-kinases; PKA, protein kinase A; PCOS, polycystic ovary syndrome; PRP, platelet-rich plasma; RFS, recurrence-free survival; ROS, reactive oxygen species; TMA, tissue microarray.

## Dysregulation of STC in Gynecological Pathologies

### Implantation Failure Linked to Impaired Endometrial Decidualization

Embryo implantation is a highly intricate process involving precise coordination between the maternal endometrium and the embryo to achieve a successful pregnancy (89, 90). Endometrial decidualization, characterized by significant morphological and functional changes in endometrial stromal cells (eSCs) during implantation, is orchestrated by a complex interplay of transcription factors, cell cycle regulators, and signaling pathways induced by progesterone (P4) and/or cyclic adenosine monophosphate (cAMP) (91, 92). Impaired decidualization has been implicated in various gynecological pathologies and pregnancy complications, including infertility, recurrent miscarriages, and uteroplacental dysfunctions (93-95).

While *STC1* null mice exhibit fertile and normal growth, indicating its dispensable function in mouse reproduction and growth (96), extensive evidence underscores its pivotal role in human embryo implantation. Transcriptome data from high-throughput studies have confirmed *STC1* expression, being a well-studied P4-responsive gene, throughout the menstrual cycle, with a notable peak observed during the midsecretory phase compared with the proliferative phase (68, 97, 98). These data revealed the dynamic expression of STC1 across the endometrial epithelium, stroma, and uterine fluid at both gene and protein levels, following the menstrual cycle phases. Notably, Albani et al documented that along with matrix metalloproteinase (MMP3, 7, 10, and 11), *STC1* stands out as a specific mRNA marker in menstrual fluid, consistently detected in endometrial tissue samples throughout the menstrual cycle (99). Taken together, these observations highlight the significance of *STC1* as an endometrial receptivity gene during the window of implantation (WOI), potentially positioning STC1 as a biomarker of successful implantation (100-102). Given the consistent homogeneous expression of *STC1* during the WOI in patients who undergo successful in vitro fertilization (IVF) treatment, *STC1* has been reported as an endometrial receptivity marker gene for successful implantation (66, 103). Moreover, Kuroda et al reported *STC1* to be one of the top upregulated genes in undifferentiated human eSCs upon treatment with platelet-rich plasma (PRP), suggesting its potential role in reprogramming cellular processes influenced by PRP infusion involved in embryo implantation during IVF treatment (67). In addition to STC1, STC2 has also been identified as a key gene in the predictive model for live birth outcomes, indicating its importance in molecular mechanisms of successful embryo implantation, decidualization, and subsequent live birth following assisted reproductive techniques (104-107). All these data suggest that elevated expressions of both STC1 and STC2 play a pivotal role in the molecular mechanisms underlying the decidualization process required for implantation.

### Pregnancy-Related Complications and Developmental Disorders

Despite the abundance of data on STC concerning the human endometrium, few data exist regarding their expression in the human placenta. Initially, Uusküla et al identified elevated *STC1* expression in the human placenta from early to midgestation followed by a decreasing trend toward full term (72). Moreover, the increased *STC1* expression in patients with preeclampsia raises questions about the relevance of placental genes in disease progression and points to a possible function for STC1 as a biomarker of pregnancy complications (72). As observed in other research, high *STC1* expression may contribute to the remodeling of spiral arteries during pregnancy, hence facilitating increased nutritional and oxygen delivery to the fetus (41). Interestingly, a clinical study discovered significantly higher STC1 levels in the plasma of women with preeclampsia than in those without the condition. Additionally, genetic research has confirmed the associations between specific *STC1* gene variants and maternal hormone levels, with implications for the development of late-onset preeclampsia (39). In line with these data, Abid et al revealed syncytiotrophoblast and cytotrophoblast cells as major sites of STC1 protein localization in addition to placental endothelial and stromal cells in the first-trimester maternal placenta. This study also confirmed that cAMP signaling cascades and low oxygen levels enhance STC1 secretion, possibly via the cAMP/PKA/PI3-Kinase/Akt/SGK-1 pathway. This finding may suggest a potential protective role of STC1 against prolonged placental hypoxia in preeclampsia conditions (40). Unlike STC1, STC2 is primarily located in syncytiotrophoblast and invasive cytotrophoblast cells of human placentas, with increased levels in the basal plate compared with chronic villi. However, no significant difference in STC2 expression between pregnancies with or without preeclampsia has been found (108).

Pregnancy-associated plasma protein A (PAPP-A) and PAPP-A2 are highly expressed genes in the human placenta and are detected in maternal blood during gestation (109). The altered expression of these proteins has been linked to multiple pregnancy-associated complications, including preeclampsia, fetal growth restrictions, and metabolic derangements (110-112). Research data have also linked STC expression to PAPP-A and PAPP-A2 proteins, crucial regulators of IGF bioavailability, essential for normal fetal development (113, 114). The enzymatic activity of PAPP-A and PAPP-A2 is predominantly downregulated by STC2 and also occasionally by STC1, indicating their correlation with the peripheral IGF axis in newborns and during development (28, 115-117). Indeed, transgenic mice expressing human STC2 are 45% smaller than their wild-type counterparts (118), possibly via inhibitory interaction between STC2 and PAPP-A, leading to the breakdown of IGF-binding protein 4 (IGFBP4) and consequently reduced IGF signaling (27, 118). Furthermore, genetic studies have also identified a link between PAPP and STC as the cause of short stature (119, 120). Additionally, research data indicate that STC plays a pivotal regulatory role in mammalian IGF activity by proteolytic inhibition of PAPP-A during human ovarian follicular development, underscoring the need for further studies to elucidate the precise mechanism targeting STC during ovarian development (35, 121).

### Endometriosis

Endometriosis is an estrogen (E2)-dependent gynecological condition characterized by the presence and growth of endometrial-like tissue outside the uterus, with a prevalence of 6% to 10% among fertile-aged women but which is responsible for 30% to 50% of infertility cases (122, 123). Regarding infertility, 1 of the key questions that has been extensively addressed is whether women with endometriosis experience poor embryo quality or compromised endometrial receptivity. This debate postulates the potential role of STC in impacting both of these critical factors (124, 125).

For the first time, Aghajanova et al reported altered *STC1* expression in the endometrial epithelial and eSCs in women with endometriosis (68). In in vitro experiments, eSCs treated with cAMP, known as an inducer of STC-1 expression, demonstrate dramatically upregulated *STC1* expression in healthy eSCs but to a lesser extent in eSCs from women with endometriosis, possibly via PKA pathways. The heightened *STC1* in healthy cells confirms its involvement in the process of decidualization, which is imperative for successful implantation (126). Conversely, the reduced *STC1* level in eSCs may indicate a possible decidualization defect in women with endometriosis (68). Of note, *STC1* expression is not significantly affected in eSCs decidualized with E2 and P4 in either the healthy or endometriosis group, suggesting that sex steroids do not regulate endometrial *STC1* expression (68), consistent with findings from the equine endometrium (37). In addition, the upregulation of the STC1 protein in endometrial fluid in the secretory phase compared with the proliferative phase has been observed in healthy women compared with women with endometriosis. The high abundance of STC1 in the secretome of the receptive endometrium from women without endometriosis may indicate a greater likelihood of successful implantation (68). Interestingly, transcriptomic data have revealed the restoration of *STC1* gene expression following surgical intervention to relieve symptoms and promote fertility in patients diagnosed with moderate to severe stages of endometriosis, highlighting its critical role in impacting endometrial receptivity (127). Given the significant role of calcium homeostasis in human embryo receptivity and implantation (128-130), the association between STC1 and the calcium-sensing receptor (*CASR*) was also investigated in these settings. However, *CASR* was not detected in endometrial cells and its levels did not exhibit significant differences in women with or without endometriosis (68). These findings may support the notion that STC1 acts via paracrine signaling, similar to growth regulation and cancer development (58, 131).

### Polycystic Ovary Syndrome

Polycystic ovary syndrome (PCOS) is a complex endocrine and metabolic disorder affecting 4% to 20% of women of fertile age, depending on demography and diagnostic criteria (132, 133). This multifaceted syndrome is characterized by irregular menstrual cycles, excessive androgen levels, ovarian dysfunction, insulin resistance, chronic inflammation, and impaired glucose metabolism (134). In addition, emerging data suggest that endocrine dysfunction, low-grade inflammation, and vitamin D deficiency adversely affect bone metabolism in these women (135). Moreover, women with PCOS experience subfertility, compromised endometrial receptivity, pregnancy complications, and an increased risk for developing endometrial cancer (EnCa) (69, 136, 137). Given the critical role of *STC1* as an endometrial receptivity marker (66), and its involvement in modulating hypoxic and inflammatory responses as a prosurvival factor (138, 139), it is postulated that dysregulated STC1 expression may negatively affect the likelihood of successful pregnancy and live birth rates in women with PCOS.

Data obtained from our previous study utilizing endometrial biopsy samples demonstrated increased STC1 levels in the secretory phase in eSCs from non-PCOS women and those with PCOS (69). However, this pattern was inconsistent among younger women with a severe PCOS phenotype and higher body mass index (BMI), which is potentially indicative of an eSC defect in women with PCOS (69). Further investigation of eSCs treated with cAMP showed significant upregulation of *STC1* only in women without PCOS. Interestingly, since *STC1* was not directly induced by steroid hormones E2 and P4, reduced basal PKA activity was demonstrated in women with PCOS, suggesting defective cAMP-mediated PKA signaling in the PCOS endometrium (69). Furthermore, *STC1* levels were also significantly higher under hypoxic conditions in healthy women than in women with PCOS, suggesting a plausible defense mechanism against the adverse effect of hypoxia. This altered gene expression appears to be associated with an attenuated response to endometrial stress, suggesting a potential defective mechanism in women with PCOS. However, elevated expression of *STC1* presented in women with obesity or overweight compared with those with a lower BMI, suggesting that obesity may be a stress factor that leads to the induction of endometrial *STC1* expression. However, this effect was not observed in women with PCOS. Despite a 3-month lifestyle intervention, commonly recommended as the first-line therapy for women with PCOS and obesity, *STC1* expression in the PCOS endometrium remained unchanged, suggesting impaired regulation of STC1 in PCOS regardless of BMI (69). Due to the limited data, it is difficult to conclude the origin of altered STC in women with PCOS. Elevated expression of STC could potentially stem from dysregulated bone metabolism, vitamin D deficiency, and altered parathyroid hormone dynamics evident in renal proximal tubular cells (140).

### Uterine Polyps and Abnormal Bleeding

Abnormal uterine bleeding, often coexisting with polyps and fibroids, is a common pathologies with an unknown etiology affecting female reproductive health (141). Mass spectrometry data from Shapiro et al showed the upregulation of *STC1* in eSCs treated with thrombin or hypoxic conditions, which was observed in women experiencing progestin-only contraceptive-induced abnormal uterine bleeding (70). The elevated expression under treatment conditions prompts consideration of whether this overexpression stems from the disease itself or therapeutic intervention. However, this overexpression of *STC1* is further supported by microarray data from biopsy samples, which suggest that *STC1* negatively impacts angiogenesis and vessel integrity (70, 142). While this finding highlights the role of STC1 as a potential target for mitigating abnormal uterine bleeding, the scarcity of data prevents a clear understanding of its precise role in angiogenesis within the context of the human endometrium (10, 143).

Furthermore, approximately 32% of infertile women undergoing IVF are diagnosed with endometrial polyps, characterized by excessive growth of endometrial glands extending into the uterine cavity, suggesting a potential causative relationship between these polyps and infertility (144, 145). In a recent case–control study involving 49 idiopathic infertile women, including those both with and without polyps, the researchers observed significantly higher STC1 and STC2 levels in endometrial biopsy tissues among those patients with polyps. This study highlighted the role of STC in the development of endometrial polyps, thereby impacting endometrial receptivity and potentially contributing to unexplained infertility in these women (71). Interestingly, transcriptomic data analysis conducted by Chiu and colleagues did not observe significant differential expression of either of the *STC* genes in patients with endometrial polyps (146). These analyses indicate inconsistent gene and protein expression levels, which may suggest potential unidentified post-transcriptional regulatory mechanisms modulating STC1 and STC2 mRNA translation, commonly observed in endometrium research (68, 69).

## Dysregulation in Gynecological Cancers

### Ovarian Cancer

Ovarian cancer (OvCa) is the second most lethal gynecological malignancy, with approximately 239 000 new cases and 152 000 fatalities reported worldwide annually (147, 148). The majority of patients experience poor prognosis, with over 75% diagnosed at an advanced metastatic stage, resulting in a global 5-year relative survival rate typically falling between 30% and 40% (149, 150). The high mortality and poor prognosis associated with OvCa present significant challenges for clinicians, stemming from delayed diagnosis, advanced metastasis, and resistance to chemotherapy (151).

With substantial experimental evidence, *STC1* was initially identified as an HIF-1 target gene in OvCa cell lines, playing a pivotal role in reprogramming ovarian tumor metabolism, unlike other cancers (53). Significantly elevated STC1 protein level in serum and tissue samples from patients with OvCa, including cell lines, suggests its role as a potential diagnostic biomarker (74, 75). This heightened expression is in turn associated with aggressive OvCa progression, influencing proliferation, migration, cell cycle regulation, and antiapoptotic processes (75). Moreover, Yang et al demonstrated that elevated *STC1* in the malignant stroma modulates the tumor microenvironment, promoting metastasis through EMT and Akt phosphorylation (55). Another study by Bajwa et al uncovered increased STC1 localization in mesothelial cells, the primary site of metastasis, evident in both ex vivo and in vivo conditions (76). In fact, elevated *STC1* in conjunction with angiopoietin-like 4 (ANGPTL4) directly modulates the tumor microenvironment, enhancing aggressive OvCa progression (76). In addition, single-cell RNA sequencing data also confirmed the prominent role of *STC1* in advanced peritoneal metastasis, lipid metabolism, and resistance to cisplatin chemotherapy, possibly through the integrin β6 (ITGβ6)/PI3K/Forkhead box C2 (FOXC2) signaling axis in vitro (77). Based on these findings, STC1 is considered a potential therapeutic target, particularly for patients with cisplatin chemoresistant OvCa (77). Sevoflurane, an anesthetic, has shown favorable effects in inhibiting tumor proliferation, invasion, and migration in various tumors, including glioma, lung, colon, and EnCa, by downregulating *STC1* expression (152, 153). This downregulation reduces migration and invasion while enhancing apoptosis via Akt/mTOR/p70S6k signaling pathways and MMP9 activity. Consistently, overexpression of *STC1* reversed this inhibitory effect in OvCa models both in vivo and in vitro, elucidating the potential of sevoflurane as an anticancer drug targeting STC1 activity (78).

Despite the contradictory regulatory role of STC in cancer progression, a study by Law et al indicated the role of STC2 as a positive regulator in OvCa progression in vitro (62). As an HIF-1 target gene, STC2, when overexpressed, induces EMT under hypoxic conditions, consequently enhancing migration and invasion. These metastases are possibly mediated via ERK1/2 signaling pathways along with heightened levels of reactive oxygen species in OvCa in vitro models (79). A later retrospective cohort study also validated these prior findings, demonstrating that high-grade serous cancer, a lethal form of OvCa, exhibited remarkedly increased STC2 levels, positively correlating with clinicopathological factors and poor OS (80). Gene expression analysis also confirmed a direct association between STC2 and high motility gene group A2 (HMGA2) genes, contributing to aggressive OvCa progression by promoting the EMT process (80). Taken together, these data indicate the prognostic role of both STC proteins in OvCa progression.

### Endometrial Cancer

EnCa is the sixth most common gynecological malignancy, primarily localized in the epithelium of the uterine inner lining, with over 400 000 new cases and over 800 000 deaths per year globally (154, 155). Critical risk factors for EnCa include altered steroid receptors, inflammation, obesity, family history, and older age (156). Type I EnCa, often low-grade and E2-driven, is prevalent in premenopausal or perimenopausal women. In contrast, type II EnCa, a more aggressive nonendometrioid tumor, is common in postmenopausal women, independent of E2 levels, aligning with the high copy number molecular subtypes (157).

Despite the presence of *STC1* in gene expression data (158), its role in EnCa progression remains unclear. In our previous study with a tissue microarray cohort of hysterectomy specimens from 832 EnCa cases, 99.15% of the cases showed positive STC1 staining primarily in the endometrial epithelium, highlighting potential tumor microenvironment modulation via the EMT process (81). Interestingly, decreased STC1 expression was linked to aggressive clinicopathologic features such as high-grade tumors, deep myometrial invasion, lymphovascular space invasion, and large tumor size. Despite the moderate association between low STC1 and the DNA mismatch repair deficiency subgroup, no association has been reported with disease-specific survival, suggesting a protective role for STC1 in EnCa progression, conflicting with findings from other studies (77, 159, 160). Furthermore, weak STC1 expression was also observed in women with obesity and type 2 diabetes mellitus who also had EnCa (81). Consistent with our data, elevated STC1 expression has been observed in low-grade compared with high-grade endometrioid EnCa, suggesting a potential role of STC1 as a tumor differentiation marker (82).

Regarding STC2, Aydin et al reported the positive staining of STC2 protein expression with a prevalence of 73.5% in endometrioid-type EnCa samples. However, increased STC2 expression was significantly linked to grade 2 to 3 tumors and to an increased likelihood of disease recurrence (83). Moreover, their multivariate analysis data highlighted both STC2 expression and tumor grade as independent predictors of disease recurrence. While EnCa samples with high STC2 expression exhibited significantly poorer recurrence-free survival (RFS), OS remained the same regardless of STC2 expression levels. These findings underscore the elevated role of STC2 expression as a negative prognostic factor, suggesting a heightened risk of recurrence in endometrioid EnCa (83).

However, none of the above-mentioned studies explored the underlying mechanism or signaling pathways linked to the involvement of STC1/STC2 in EnCa progression. A recent study by Wang et al, however, did provide mechanistic insights into the critical role of STC2 in EnCa progression (84). The study identified *STC2* as an E2-responsive gene, similar to findings for breast cancer (161). According to their data, type I E2-dependent EnCa tissues showed a higher expression of STC2 than type II E2-independent EnCa tissues. In addition, E2 treatment increased *STC2* expression by promoting cellular proliferation and inhibiting apoptosis in EnCa cell lines (84). Conversely, *STC2* knockdown reduced cell viability and proliferation while promoting apoptosis in E2-treated cell lines. Furthermore, the loss of *STC2* suppressed E2-stimulated tumor growth in vivo, suggesting that STC2 deficiency inhibits E2-stimulated proliferation and tumor growth by activating phosphorylated-AMP–activated protein kinase (AMPK) signaling, particularly in type I EnCa (84). These findings may validate the correlation between STC and hormonal regulation in EnCa progression.

### Cervical Cancer

Cervical cancer (CeCa) ranks as the fourth most common cause of malignancy and mortality among women worldwide and is among the top 3 malignancies affecting reproductive women under 45 years of age, with variations depending on demographic factors (162, 163). The prognosis for CeCa is poor due to a lack of understanding of the underlying cellular mechanisms in advanced metastatic or recurrent stages (164). In CeCa, the progression from cervical intraepithelial neoplasia to malignancy is linked to the persistent infection of human papillomavirus (HPV) (165).

In contrast to OvCa, STC1 exhibits downregulation in tissues diagnosed with CeCa compared with noncancerous cervical tissue (15). This downregulation correlates with increased cell growth, migration, and invasion when *STC1* is knocked down, while overexpression negatively regulates such cellular activities in CeCa cell lines. Furthermore, the interaction of the NF-κB p65 protein directly bound to the *STC1* promoter activates the expression of STC1 in CeCa cells, indicating suppressed cell proliferation and invasion through NF-κB p65 activation (15). A follow-up study by this team revealed that heightened STC1 expression facilitated cellular apoptosis via the NF-κB phosphor-P65 (Ser536) pathway, regulated by PI3K/AKT, IκBα, and IKK signaling cascades. Conversely, silencing *STC1* was found to attenuate the proliferation of both in vivo and in vitro models (85). In addition, decreased *STC1* expression was noticed in advanced stages of CeCa, validating the notion that low *STC1* expression is a marker of advanced-stage disease (85). Interestingly, the CeCa cell line treated with trichostatin A, an anticancer drug, showed significantly high *STC1* levels with accelerated rates of apoptosis and autophagy (86). Moreover, STC1 plays a critical role in controlling the PRMT5/STC1/TRPV6/JNK axis in trichostatin A–mediated effects on CeCa cells, as evidenced by the increase in transient receptor potential cation channel-subfamily V-member 6 (TRPV6) and the decrease in p-JNK protein levels upon STC1 inhibition (86).

Intriguingly, *STC2* expression was reported to be much higher in tumors of patients with CeCa than in surrounding normal cervical tissues. Elevated *STC2* expression is associated with shorter OS, whereas lower expression correlates with longer OS and progression-free survival after radiotherapy. Moreover, increased *STC2* expression is also linked to lymph node metastasis, indicating its role as a prognostic marker in postradiotherapy follow-up in patients with CeCa (87). Consistent with these data, findings from another study also reported high STC2 expression and a positive correlation between STC2 and cellular proliferation in tissue and cell lines. Elevated STC2 levels were also reported in cisplatin-resistant CeCa cell lines, indicating resistance to platinum-based chemotherapy drugs in vitro. Additionally, silencing or overexpressing *STC2* modulated cellular proliferation and apoptosis. Conclusively, the team also discovered the pivotal regulatory role of STC2 involving MAPK signaling pathways between cisplatin-sensitive and resistant CeCa cells (88).

## Transcriptomic Database and Bioinformatic Tools for the Predictive Value of *STC*

The Cancer Genome Atlas (TCGA) offers a comprehensive genomic and proteomic molecular landscape of various cancer types, including OvCa, EnCa, and CeCa (166). Analyzing these multidimensional, diverse data necessitates sophisticated computational and bioinformatics tools. Fortunately, several web-based tools are available to assist researchers in understanding the diagnostic and prognostic implications of STC in gynecological cancers using TCGA data. Moreover, considering HPV infection as the primary risk factor for the majority of CeCa cases, including other anogenital carcinomas (167), the TCGA may offer a unique opportunity to explore the potential link between HPV infection and STC protein expression through multivariable survival analysis and explainable artificial intelligence (168-170).

For instance, platforms like cBioPortal and UCSC Xena allow for an interactive exploration of multidimensional cancer genomic datasets, facilitating comprehensive analyses of genetic alterations, gene expression, and clinical data (171, 172). Furthermore, KMplotter and GEPIA/GEPIA2 can provide crucial insights into the correlation between gene expression and patient survival, aiding in the assessment of the prognostic significance of *STC* gene expression (173, 174). Additionally, tools like the UCSC Cancer Genomics Browser and UALCAN/UALCAN2 offer sophisticated visualization and analysis capabilities, allowing researchers to better understand *STC* gene alterations within the context of cancer phenotypes (175, 176). Furthermore, GENI and MEXPRESS enable gene set enrichment analysis and visualization of expression, DNA methylation, and clinical data, further enriching our understanding of STC biology (177, 178). Lastly, ExplORRNet integrates miRNA expression analysis and patient survival assessments, shedding light on the intricate regulation of STC and its prognostic implications in gynecological and other cancers (179). By leveraging these diverse and powerful bioinformatics tools, researchers can explore the diagnostic and predictive roles of *STC* across various cancer types, utilizing the wealth of information provided by these cancer databases.

**Figure 2.**
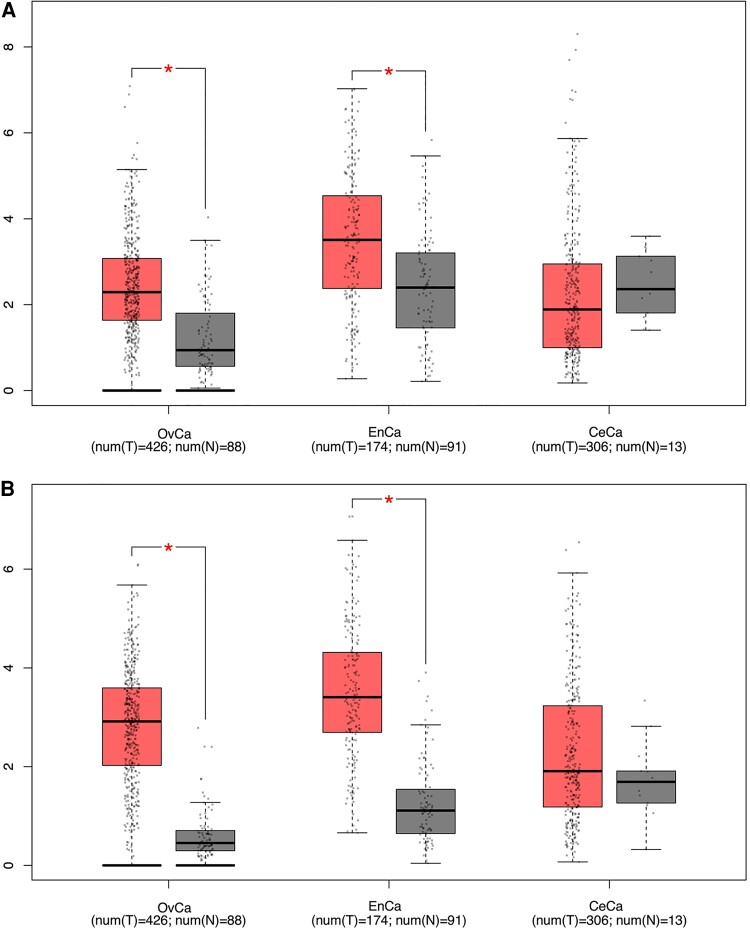
TCGA-based gene expression levels of *STC1* and *STC2*. (A) *STC1*; (B) *STC2* in OvCa, EnCa, and CeCa cancers. The number of tumor tissues (T) Is represented by red bars, while the number of normal tissues (N) Is shown in grey bars. A red star indicates statistical significance with *P* < .05. Abbreviations: OvCa, ovarian cancer; EnCa, endometrial cancer; CeCa, cervical cancer. Generated using GEPIA website.

To demonstrate the utility of the aforementioned tools, we analyzed *STC1* and *STC2* expression and clinicopathological characteristics in OvCa, EnCa, and CeCa using web-based tools, including GEPIA and KMplotter (173, 174). Since the number of normal tissues available for gynecological cancer comparisons in TCGA was insufficient, normal tissues were included as a reference from the Genotype-Tissue Expression (GTEx) resource (180). Our gene expression analysis revealed significant upregulation of both *STC1* and *STC2* in OvCa cases (*P* < .05), underscoring their potential relevance in these malignancies, in line with previous research (Fig. 2) (50, 77). Contrary to the literature (81, 82), both *STC1* and *STC2* expression were found to be significantly higher in EnCa cases than endometrial tissue without tumors. On the other hand, *STC1* showed a downward trend in CeCa (Fig. 2A) (15, 85), while *STC2* displayed higher expression patterns, consistent with the literature (Fig. 2B) (87, 88). However, no statistical significance was observed for either *STC* for CeCa (Fig. 2).

**Figure 3.**
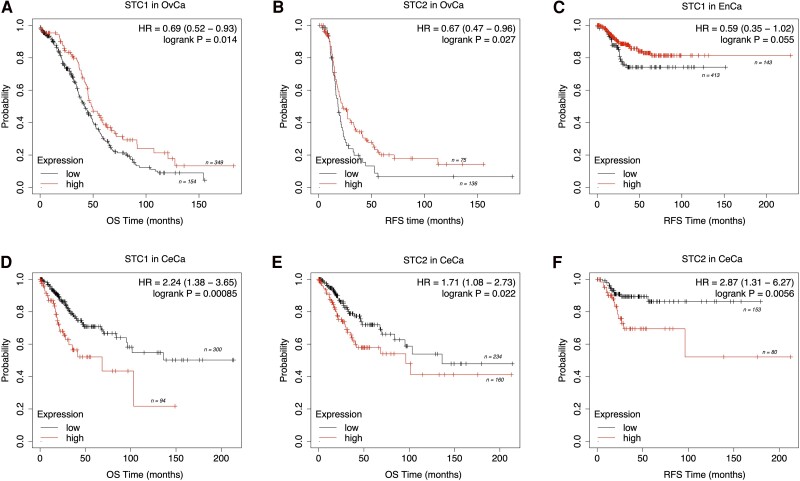
TCGA-based Kaplan–Meier survival plots of *STC1* and *STC2*. (A) OS of *STC1* in OvCa; (B) RFS of *STC2* in OvCa; (C) RFS of *STC1* in EnCa; (D) OS of *STC1* in CeCa; (E) OS of *STC2* in CeCa; (F) RFS of *STC2* in CeCa cases. *STC* expression levels are shown as high (red) and low (black) accompanied by the number of cases (n). The median follow-up for OS and RFS was 200 months for both EnCa and CeCa, while that for OvCa was 150 months due to data availability. Abbreviations: OS, overall survival; RFS, recurrence-free survival; OvCa, ovarian cancer; EnCa, endometrial cancer; CeCa, cervical cancer; HR, hazard ratio; statistical significance with *P* = .05. Generated using KM plotter website.

Finally, our findings revealed significant associations between *STC* gene expression and survival outcomes of patients. While a thorough survival analysis utilizing TCGA was conducted for all 3 cancer types, only statistically significant associations (*P* ≤ .05) for survival are depicted and discussed in this review (Fig. 3). In contrast, to demonstrate the distinct expression patterns of *STC1* and *STC2* across all 3 cancer types, nonsignificant associations (*P* > .05) for survival are presented elsewhere (Fig. S1 (181)). In OvCa, *STC1* expression showed a significant association with OS, with lower expression levels correlating with reduced OS (hazard ratio [HR] 0.69, 95% CI 0.52-0.93, *P* = .014) (Fig. 3A), whereas lower *STC2* levels were associated with longer RFS in OvCa (HR 0.67, 95% CI 0.47-0.96, *P* = .027) (Fig. 3B). In EnCa, although the association between *STC* expression and RFS outcomes did not reach statistical significance (HR 0.59, 95% CI 0.35-1.02, *P* = .055), the findings were close to significance, suggesting potential prognostic implications for EnCa, with lower *STC1* expression associated with shorter RFS time (Fig. 3C). Thus, further investigation is needed to understand the precise prognostic role of STC in EnCa. In CeCa, higher *STC1* expression was correlated with reduced OS (HR 2.24, 95% CI 1.38-3.65, *P* = .00085) (Fig. 3D). On the other hand, although STC2 expression in CeCa showed a significant association with OS time (HR 1.71, 95% CI 1.08-2.73, *P* = .022), the interpretation of this association requires caution, as the difference in expression cannot be clearly observed by the survival curve (Fig. 3E). However, the higher expression of *STC2* in CeCa is correlated with longer RFS time (HR 2.87, 95% CI 1.31-6.27, *P* = .0056) (Fig. 3F). Overall, our TCGA-based expression and survival analysis highlights the potential of *STC* gene expression as a prognostic marker in CeCa and OvCa (20, 47, 65).

## Conclusion, Limitation, and Future Perspectives

STC proteins 1 and 2 function as paracrine factors in humans, playing multifaceted roles across various physiological and pathological processes. This review highlights the challenges posed by the ubiquity and contradictory role of STC1 and STC2 in gynecological and malignant conditions, which complicate their interpretation and limit their potential as biomarkers. Given their ubiquitous differential expression of STC1 and STC2, further investigation is warranted to elucidate their precise involvement in female reproduction, infertility, and pregnancy-associated pathologies, including implantation failure, endometriosis, PCOS, uterine polyps, compromised endometrial decidualization, abnormal placental development, and preterm birth in larger clinical study settings. However, dysregulation of these proteins in both tissue and circulation in gynecological endocrine conditions with comorbidities such as diabetes and obesity complicates the attribution of these alterations specifically to gynecological pathologies or associated issues. Given the fluctuating expression of STC across the menstruation cycle, future studies could focus on well-stratified disease groups, including menopausal women, to gain deeper insights into STC regulation. In menopausal women, drastic changes in calcium metabolism could impact STC expression levels, which might also vary with conditions such as diabetes, bone pathologies, or obesity. Understanding this dysregulation is crucial, as it may also act as a defense mechanism against the adverse effects of these pathologies including cancer.

To explore the potential ubiquitous role of STC proteins as diagnostic and prognostic biomarkers in gynecological conditions, artificial intelligence-directed advanced histology technologies and in vitro disease models using induced pluripotent stem cells may offer promising opportunities over traditional histology and animal models respectively, particularly for complex diseases such as endometriosis and PCOS (182-184). Given the contradictory role of STC in cancer progression depending on tissue type, more extensive studies are needed in the context of gynecological cancers, involving larger patient cohorts and comprehensive clinical and genetic characterization. In the current review, we took preliminary steps by utilizing novel bioinformatic tools encompassing public cancer data repositories, like the TCGA, to investigate the importance of *STC1* and *STC2* gene expression in gynecological cancers in predicting patient prognosis. To date, data highlighting the pivotal role of STC in gynecological cancers are scarce. By utilizing intuitive bioinformatic tools and public repositories, researchers can continue to explore and understand the diagnostic and predictive values of *STC* genes across various cancer types, leading to improved patients’ outcomes in gynecological malignancies.

## Search Strategy

A search of the PubMed database was conducted for articles in the English language using the advanced search builder tool, focusing on the following key terms “stanniocalcin,” “STC1,” “STC2,” “female reproduction,” “endometrium,” “endometriosis,” “polycystic ovary syndrome,” “polyps,” “preeclampsia,” “pregnancy complications” “implantation,” “decidualization,” “ovarian cancer,” “endometrial cancer,” “cervical cancer,” “bioinformatic tools,” and “the cancer genome atlas.” The selection criteria were meticulously applied by the authors after being carefully analyzed regarding their relevance, importance, and impact. References were also sourced directly from the articles included in this manuscript.

## Data Availability

This article does not involve data sharing.

## Abbreviations

BMI: body mass index
cAMP: cyclic adenosine monophosphate
CASR: calcium-sensing receptor
CeCa: cervical cancer
E2: estrogen
EMT: epithelial–mesenchymal transition
EnCa: endometrial cancer
eSC: endometrial stromal cell
HIF: hypoxia-inducible factor
HPV: human papillomavirus
IGF: insulin-like growth factor
IVF: in vitro fertilization
MMP: metalloproteinase
OS: overall survival
OvCa: ovarian cancer
P4: progesterone
PAPP: pregnancy-associated plasma protein
PCOS: polycystic ovary syndrome
PRP: platelet-rich plasma
RFS: recurrence-free survival
STC: stanniocalcin
TGGA: The Cancer Genome Atlas
WOI: window of implantation
